# Single-Port Fluorescence Immunoassay for Concurrent Quantification of Live and Dead Bacteria: A Strategy Based on Extracellular Nucleases and DNase I

**DOI:** 10.3390/molecules30061374

**Published:** 2025-03-19

**Authors:** Yuhan Wang, Han Dong, Hang Yu, Shaofeng Yuan, Hideya Kawasaki, Yahui Guo, Weirong Yao

**Affiliations:** 1 State Key Laboratory of Food Science and Resources, School of Food Science and Technology, Jiangnan University, Wuxi 214122, China; 2 Faculty of Chemistry, Materials and Bioengineering, Kansai University, Suita 564-8680, Japan

**Keywords:** foodborne pathogens, extracellular nuclease, viable bacteria detection, aptamers, immunoassay

## Abstract

Bacteria are the primary culprits of global foodborne diseases, making bacterial detection one of the most critical aspects of food safety. The quantification of viable and dead bacteria is typically achieved through distinct methodologies, such as culture-based methods and molecular biological techniques. These approaches often have non-overlapping requirements in terms of sample pre-treatment and detection equipment. However, in this presented work, bacterial extracellular nucleases and DNase I were utilized to achieve the simultaneous quantification of both live and dead bacteria in a single well of a microplate. The detection limits of the method for live and dead bacteria are estimated to be 7.13 × 10^5^ CFU/mL and 3.54 × 10^5^ CFU/mL, respectively. In the application of detecting bacteria in pickled pork stewed bamboo shoot soup, the detection limit for live bacteria can be reduced to as low as 10^2^ CFU/mL within 24 h after enrichment cultivation.

## 1. Introduction

Foodborne pathogens pose a significant threat to public health and global food safety [1,2], resulting in considerable morbidity and mortality. According to the World Health Organization (WHO), approximately 600 million people fall ill and 420,000 die annually due to foodborne diseases [3]. Fecal contamination of food and drinking water is a major route for the transmission of pathogenic bacteria to humans [4]. Common foodborne pathogens include *Salmonella* [5], *Campylobacter*, and *Listeria monocytogenes* [6,7]. Infections caused by *Salmonella* are among the most common causes of bacterial food poisoning, with *Salmonella* Typhimurium being the most frequently reported serotype [8,9]. Given the severe health consequences and significant economic losses associated with foodborne pathogens, the development of rapid and accurate detection methods is of paramount importance. Immunological methods, such as ELISA [10], are widely utilized for the rapid and specific detection of foodborne pathogens. These methods rely on antigen–antibody interactions, offering high specificity and sensitivity for pathogen detection. Given the zero-tolerance policy for these pathogens, immunological assays are essential for rapid detection, ensuring compliance with food safety regulations. Current detection methods for bacteria can be broadly categorized into the following two main types: total bacterial count (including both live and dead bacteria) and viable bacterial count.

The total bacterial count is essential for evaluating the contamination level and potential for toxin production [11]. This can be achieved through techniques such as PCR and immunological methods. For instance, X. Fan et al. [12] utilized a novel qPCR method to achieve quantitative detection of viable and non-viable lactic acid bacteria in fermented milk. S. Jain et al. [13] employed a surface-aminated polycarbonate membrane-enhanced ELISA to achieve highly sensitive detection of *S.* Typhimurium.

The viable bacterial count is critical for assessing the safety of food products over a certain period of time [14]. Traditional methodologies, such as plate culture techniques, rely on visible colony formation on solid media for enumeration. These established methods are widely employed and recognized within numerous standardized protocols; however, they suffer from drawbacks, such as low accuracy, long detection cycles, and insufficient specificity, which limit their application to rapid and precise detection [15,16]. Some new methods for detecting viable bacteria have also been reported. For instance, Kumaravel et al. [17] developed electrochemical sensors based on the ability of esterase to decompose C8-aliphatic ester groups for detecting active *Salmonella*. Sun et al. [18] introduced a method that inhibits the GOX-catalyzed reaction to rapidly and broadly detect live bacteria through bacterial glucose metabolism. Wang et al. [19] utilized lysozyme to digest and decompose *Salmonella*, releasing ATP and thus enabling the development of a specific bioluminescence sensor, which, with sample enrichment, achieves a detection limit at the single-digit CFU/mL level.

Some studies have found that many harmful pathogens can secrete extracellular nuclease [20,21], which may either be anchored to the cell envelope or completely released into the extracellular space. These nucleases play crucial roles in bacterial virulence, biofilm formation, adhesion, invasion, acquisition and degradation of extracellular DNA, and evasion from neutrophil extracellular traps (NETs) [22,23,24]. Qian et al. [25] found a 5′-nuclease in the extracellular protein of *S*. Typhimurium, which enables the bacterium to evade macrophage-mediated extracellular killing; thus, we utilized *S*. Typhimurium in our research. Liao et al. [26] clearly proved that the extracellular protein of *S*. Typhimurium has deoxyribonuclease activity. Four protein bands with nuclease activity were found between 26–72 kDa molecular weights. Furthermore, Machado et al. [27] used the nuclease activity of *S*. Typhimurium as a biomarker of infection for the first time. The optimal substrate oligonucleotide sequence was screened, and the detection limit for *S*. Typhimurium was 10^4^ CFU/mL. Barbau-Piednoir et al. [28] divided bacterial states into four categories. The “viable but non-culturable” (VBNC) bacteria are defined as metabolically active, with an intact cell-wall/membrane, but they could not be cultured to produce colony-forming units (CFU). There are still extracellular enzymes secreted before their death in the cell membrane and living environment of such bacteria, so they still threaten biological health.

Due to differences in technical principles, the detection steps and equipment for live and dead bacteria are difficult to overlap. Live bacteria detection often relies on methods based on metabolic activity, membrane integrity, or growth, which require conditions that maintain cell viability. In contrast, dead bacteria detection typically depends on membrane damage or DNA degradation and may involve harsh conditions. Thus, almost no reports have achieved a simultaneous quantitative analysis of live and dead bacteria within a single detection process; however, in this study, we used antibodies as capture elements and gold nanoparticles modified with *S.* Typhimurium aptamers (Apt.-SNA) as signal elements, combining the two to form a probe. We then utilized extracellular nucleases and a tool enzyme (DNase I) to achieve the separate quantitative detection of live and dead bacteria within a single well of an enzyme-linked immunosorbent assay (ELISA) plate. The extracellular nuclease was employed to detect the fluorescence emitted by viable bacteria (in a broad sense), whereas DNase I was used to detect the fluorescence associated with dead bacteria.

## 2. Results and Discussion

### 2.1. Characterization of AuNPs and Apt.-SNA

**Figure 1:**
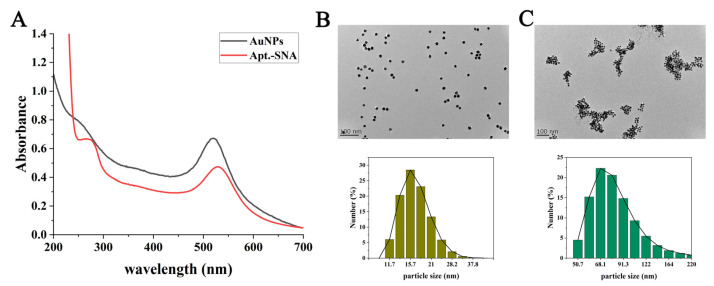
Characterization of AuNPs and Apt.-SNA: (**A**) UV-visible spectrum; (**B**) TEM image and DLS particle size distribution image of AuNPs; (**C**) TEM image and DLS particle size distribution image of Apt.-SNA.

The AuNPs prepared by the citrate reduction method of HAuCl_4_ had good dispersity. The diameter was about 15 nm. In Figure 1A, AuNPs presented the maximum absorption wavelength, at 518 nm. The maximum UV absorption peak of Apt.-SNA synthesized by the freezing method had a small degree of redshift and showed a DNA characteristic peak at 260 nm. In Figure 1B,C, TEM and DLS images showed that Apt.-SNA was slightly aggregated, and the hydrated particle size increased to about 68 nm, indicating that the aptamer chain had been successfully attached to the AuNPs’ surface. The number of aptamers attached to the SNA surface was determined by the ME substitution method to be 145 strands/NP.

### 2.2. Linear Curve of Bacterial Colony Number and Absorbance

**Figure 2:**
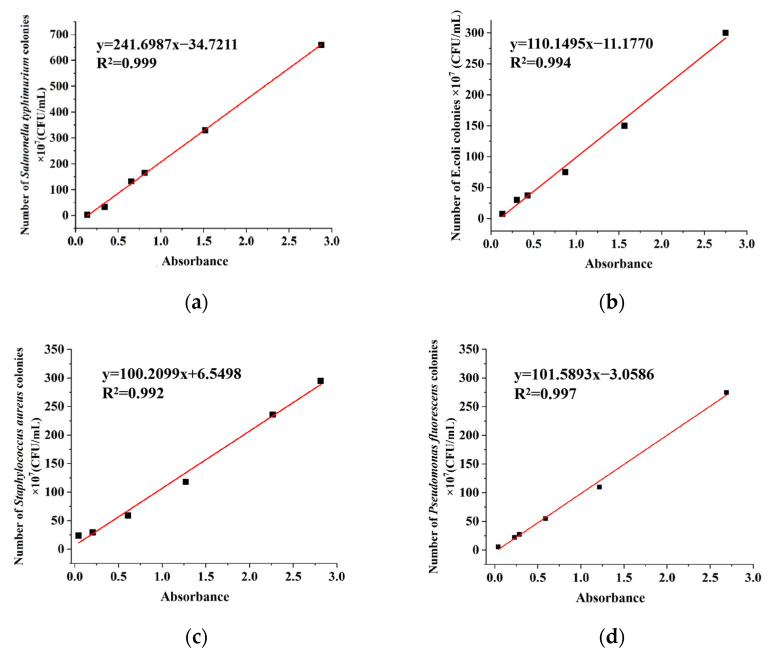
Absorbance—CFU standard curve: (**a**) *S*. Typhimurium, (**b**) *E. coli*, (**c**) *S. aureus*, (**d**) *P. fluorescens*.

In Figure 2, the relationship between the colony-forming unit (CFU) of *S*. Typhimurium, *Escherichia coli*, *Staphylococcus aureus*, and *Pseudomonas fluorescens* and absorbance was determined. The bacteria were cultured overnight, then washed twice with PBS, and serially diluted. The bacterial concentration was determined by the plate counting method. At the same time, the absorbance of the bacterial solution at 600 nm was measured with PBS solution as a blank control.

### 2.3. Detection Principle and Feasibility

**Scheme 1:**
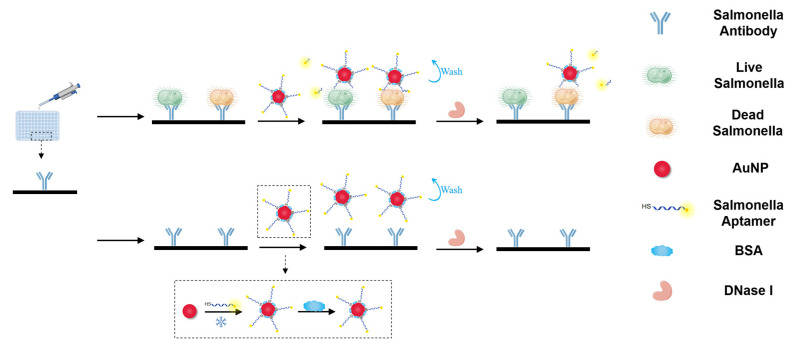
Fluorescent sensor for detection of live/dead *S*. Typhimurium.

A fluorescent detection strategy based on viable bacterial extracellular nucleases has been developed. The *S.* Typhimurium-specific aptamers connected with the FAM fluorophores, and the sulfhydryl groups at both ends were attached to the surface of the gold nanoparticles (AuNPs) through Au-S bonds to prepare the spherical nucleic acid probes (Apt.-SNA). *S*. Typhimurium antibodies were used to capture and isolate intact bacteria, and SNA can be localized and adsorbed on the surface of the bacteria. The extracellular nuclease produced by living bacteria cleaved DNA and released fluorescence (Step 1). After washing, DNase I was added to cut the probe of the surface of dead bacteria to restore the fluorescence (Step 2). Scheme 1 shows the detection process of this study. A detection scheme similar to a logic gate has been established to qualitatively and quantitatively detect viable and dead *S*. Typhimurium in food. Since live bacteria are the target of detection, their metabolic activity and viability can introduce variability, potentially affecting their detection sensitivity. In actual sample testing, pre-enrichment can be used to increase the bacterial concentration within a short period of time, thereby enhancing detection sensitivity and enabling the application of this protocol.

AuNPs can quench nearby fluorophores by static quenching and fluorescence resonance energy transfer (FRET). DNase I cleaves DNA strands internally or externally, causing the FAM fluorescent groups labeled at the DNA ends to detach from the surface of the AuNPs. The distance between the fluorophore and AuNPs increases, the FRET efficiency decreases, and the fluorescence is restored accordingly.

**Figure 3:**
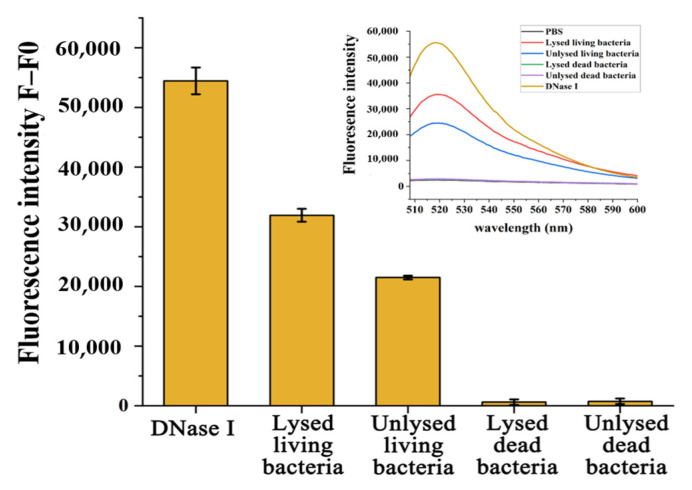
Effect of *S*. Typhimurium (living bacteria and heat-inactivated dead bacteria) on fluorescence intensity of probe.

It has been reported that nucleases in the extracellular proteins of *S*. Typhimurium cut the DNA. The newly cultured 5 × 10^8^ CFU/mL bacterial solution was incubated with a 10-fold diluted Apt.-SNA probe at 37 °C for 2 h. Figure 3 shows that the DNA strand of the probe could be cleaved by both lysed and unlysed viable bacteria, and the fluorescence could be restored. The fluorescence intensity of the dead bacteria inactivated by a high temperature did not change. This result proved that *S*. Typhimurium can produce extracellular nuclease and that intact viable bacteria can also decompose DNA to restore the probe’s fluorescence.

Since the sample solution with bacteria was centrifuged in PBS twice before detection, intracellular or extracellular nucleases released by naturally dead bacteria remained in the supernatant and did not participate in the probe reaction during the Step 1 process.

### 2.4. Optimization of Experimental Conditions

**Figure 4:**
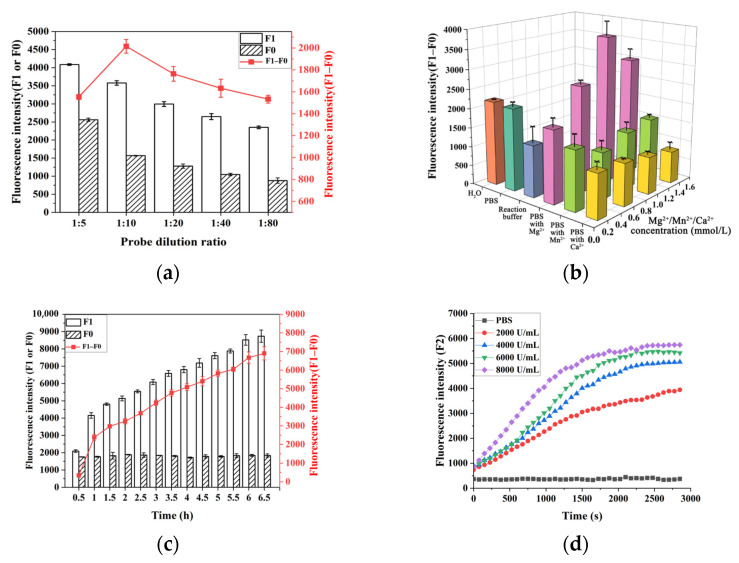
Optimization of experimental conditions: (**a**) probe dilution ratio; (**b**) extracellular nuclease reaction buffer; (**c**) extracellular nuclease reaction time; (**d**) DNase I concentration and reaction time.

The concentration of Apt.-SNA directly affects the fluorescence signal recovery effect of extracellular nuclease in viable bacteria. Too low of a concentration cannot fully absorb the nuclease digestion signal, and too high of a concentration affects the aptamer localization and increases the background signal. The dilution of the probe was set to 5, 10, 20, 40, and 80, and the Step 1 experiment of the viable bacteria detection was performed [29]. As shown in Figure 4a, initial probe fluorescence, F0, and final reaction fluorescence, F1, decreased with the decreasing probe concentration. The recovered fluorescence intensity (F1–F0) had the best effect when diluted 10 times. The dilution ratio was chosen to be 1:10.

The literature shows that the nuclease activity of *S*. Typhimurium is stable under alkalescent conditions (pH 7~10) [26]. The optimal pH for 5′-nuclease is 6.0–7.0, and the optimal temperature is 37 °C [25]. Mg^2+^, Ca^2+^, and other ions can promote nuclease activity [26], and Mg^2+^ has the best promotion effect on 5′-nuclease [25]. Based on the literature data and considering aptamer binding conditions and the optimal luminescent environment of FAM, reaction buffers of Step 1—with different ions and concentrations—were designed. As shown in Figure 4b, a PBS solution of 1 mmol/L Mg^2+^ had the best response signal.

*S*. Typhimurium extracellular nuclease can be immobilized on the outer membrane or released into the extracellular supernatant [23]. The growth retardation period of *S*. Typhimurium in the medium was approximately 2 h, and nuclease activity could be detected in the supernatant after 6 h [27]. Therefore, aptamer modified on the surface of the Apt.-SNA probe could be quickly and accurately located on the surface of the intact bacteria, and the DNA decomposition time was shortened using nucleases on the membrane. Figure 4c shows that the fluorescence of the system has a gradual increasing trend over time. Considering the signal intensity and time economy, 2 h was selected as the live bacteria’s routine enzyme digestion time. As time increases, the signal intensity and the detection sensitivity will increase.

To maximize DNase I usage efficiency and shorten the detection time, the enzymatic kinetics of DNase I were determined in Step 2 using dead bacteria as the detection object. As shown in Figure 4d, when the final enzyme concentration was lower than 6000 U/L, the final fluorescence of enzymatic cleavage could not be completely released in a short amount of time. The signal reached the plateau when the time was above 40 min. Therefore, the concentration of DNase I was 6000 U/L, and the time was 40 min.

**Figure 5:**
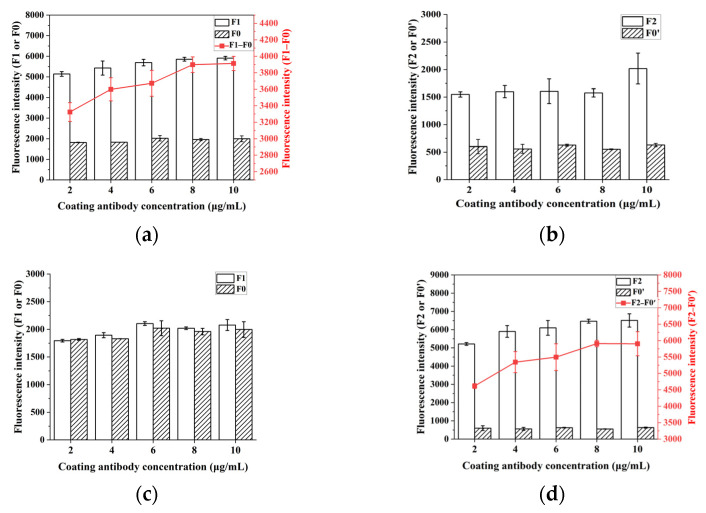
Optimization of coating antibody concentration: (**a**) Step 1, detection of viable bacteria; (**b**) Step 2, detection of viable bacteria; (**c**) Step 1, detection of dead bacteria; (**d**) Step 2, detection of dead bacteria.

The specific binding of the antigen and antibody is the key step in this method, and the concentration of the coated antigen directly affects the results of the bacterial detection effect. As shown in Figure 5, in the four types of cases, the initial signal intensities F0 and F0′ before and after probe washing were less correlated with the coating antibody concentration. The fluorescence increments in bacterial nucleases (F1–F0) and DNase I (F2–F0′) cleavage increased with increasing concentrations of coated antibodies and stabilized at 8 μg/mL. When the antibody concentration is too high, multiple antibodies may bind to one bacterium, wasting antibodies and reducing sensitivity. Too low of a concentration may fail to capture the bacteria; thus, 8 μg/mL was optimal for *S.* Typhimurium.

### 2.5. Sensitivity, Specificity, and Repeatability for the Assay

**Figure 6:**
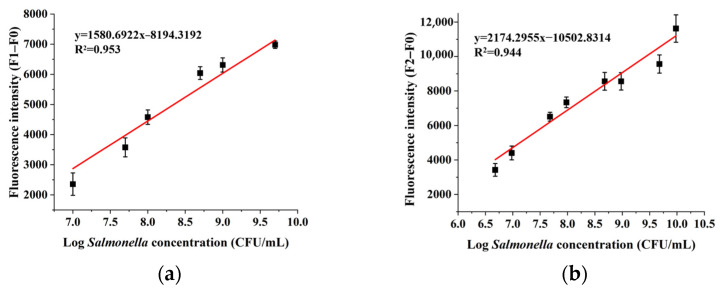
Sensitivity: (**a**) Step 1 for viable bacteria; (**b**) Step 2 for dead bacteria.

Under the above-optimized conditions, the Apt.-SNA fluorescent probe was introduced to test the detection performance of quantitative analysis methods for living and dead bacteria. In Figure 6, a linear regression equation can be established with the fluorescence enhancement value (F–F0) as the ordinate, and the logarithmic value of the *S*. Typhimurium concentration as the abscissa. Step 1 involved using nuclease from live bacteria to degrade the probes and restore fluorescence for the specific and high-concentration quantification of viable *S*. Typhimurium. The linear equation over the range of 10^7^ to 5 × 10^9^ CFU/mL was y = 1580.6922x − 8194.3192 (R^2^ = 0.953). The standard deviations of the 11 blank values were used as fluorescence signals, and the limit of detection (LOD) of viable bacteria was calculated as 7.13 × 10^5^ CFU/mL in Step 1. In Step 2, DNase I was used to cleave the probe bound to the surface of dead bacteria to restore fluorescence, enabling qualitative and high-concentration quantitative analysis of dead bacteria. In the range of 4.78 × 10^6^~9.55 × 10^9^ CFU/mL, the linear equation was y = 2174.2955x − 10502.8314 (R^2^ = 0.944). The LOD of dead bacteria in Step 2 was calculated as 3.54 × 10^5^ CFU/mL. A standard curve was constructed, with the LOD determined using *n* = 11 based on blank standard deviation and linear regression. This method enables the precise identification of *S*. Typhimurium, allowing for a one-time quantitative assessment of live and dead bacteria in enriched high-concentration bacterial suspensions.

**Figure 7:**
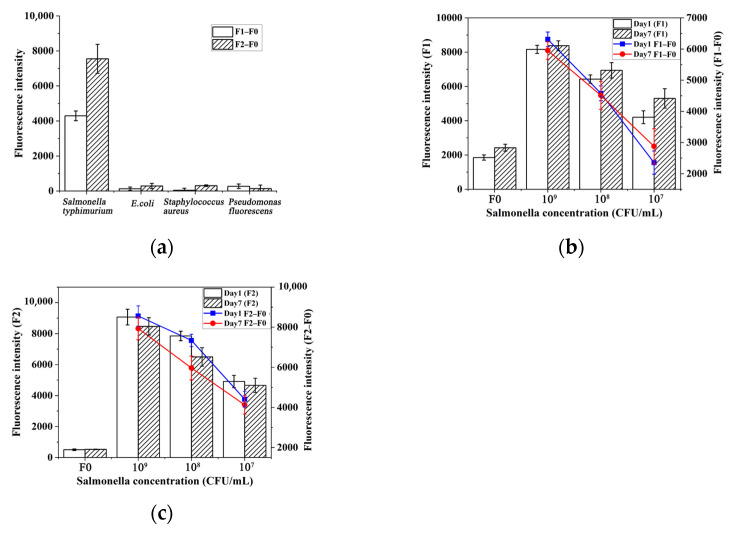
Specificity and Repeatability for the assay: (**a**) specificity; (**b**) repeatability for viable bacteria; (**c**) repeatability in Step 2 for dead bacteria.

This method combines the dual selective strategies of antigen–antibody immunological binding and aptamer adsorption. To explore the specificity of this method, three types of food-borne pathogens commonly found in foods, such as *E. coli*, *S. aureus*, and *P. fluorescens*, were selected as analytes to calculate the response fluorescence (F1–F0) of live bacteria and (F2–F0) of dead bacteria. As shown in Figure 7a, the corresponding signals of the scheme against the three pathogens were all low, which showed good specificity.

To explore the stability of the Apt.-SNA probe, the experiments were repeated on the first and seventh day of probe synthesis. For the extracellular nuclease reaction (Figure 7b), the fluorescence intensity of the blank group on the seventh day increased significantly (31%) compared to the first day. It was speculated that some DNA strands fell off the surface of AuNPs. The average change rate of F1–F0 was 9.7%, and there was still a linear relationship within the linear range. For the DNase I reaction (Figure 7c), the average decrease rate of F2–F0 on the seventh day was 10.7%, but a good signal response trend could still be obtained. Therefore, it is recommended that the probe be used within one week.

### 2.6. Soup Sample Analysis

Pickled pork stewed bamboo shoot soup was used as the practical sample. The added concentrations of live *S*. Typhimurium were 10^5^, 10^4^, and 10^3^ CFU/mL, with the simultaneous addition of dead bacteria at 10^5^ CFU/mL. The number of bacteria at different growth times was measured and compared using the plate counting method. As shown in Table 1, the blank samples had a negative signal. The initial bacterial concentration of 10^4^ CFU/mL of soup could be detected when the growth time of the sample was 8 h. The initial bacterial concentrations of 10^3^ CFU/mL and 10^2^ CFU/mL could be detected when the growth times of the samples were 16 h and 24 h, respectively.

For viable bacteria, the detection signal increased with culture time, showing results comparable to those obtained from the plate counting method. This method significantly reduces detection time while simultaneously quantifying both live and dead bacteria, providing a more comprehensive assessment of bacterial; however, this method tended to overestimate the amount of dead bacteria, likely due to some live bacteria dying during cultivation. Compared to FDA-recommended methods, our single-well detection approach simplifies the process, reducing both time and operational complexity.

## 3. Materials and Methods

### 3.1. Reagents and Instruments

Chloroauric acid (HAuCl_4_), Trisodium citrate (C_6_H_5_Na_3_O_7_·H_2_O), Polyethylene glycol 20000 (PEG 20000), Tween 20, and bovine albumin (BSA) were purchased from Sinopharm Chemical Reagents Co., Ltd. (Shanghai, China). Tris (2-carboxyethyl) phosphine (TCEP) was purchased from Innochem Technology Co., Ltd. (Beijing, China). 2-Mercaptoethanol (ME) was purchased from J&K Scientific Co., Ltd. (Beijing, China). The anti-*S*. Typhimurium antibody was purchased from the Beijing Bioss Biological Technology Co., Ltd. (Beijing, China). DNase I was extracted from bovine pancreases; the aptamer was synthesized, modified, and purified by Sangon Biotechnology Co., Ltd. (Shanghai, China) (Table 2). The aptamer sequences used in this study are from the authoritative literature [30]. *S*. Typhimurium (ATCC 14028) was purchased from Beijing Zhongke Quality Inspection Biotechnology Co., Ltd. (Beijing, China); no plasmid or antibiotic resistance genes were carried. *E. coli* (ATCC 25922), *S. aureus* (ATCC 6538), and *P. fluorescens* (ATCC 13525) were stored by the Research Center of Food Safety and Quality Control, Jiangnan University (Wuxi, China). Coating buffer (0.05 mol/L CBS at pH 9.6), dilution buffer (0.01 mol/L PBS at pH 7.2), washing buffer (0.01 mol/L PBS and 0.05% Tween 20), and 10× reaction buffer (0.1 mol/L Tris-HCl, 25 mmol/L MgCl_2_, and 1 mmol/L CaCl_2_ at pH 7.5) were used in this assay.

UV-1800 UV-Vis spectrophotometer and RF-6000 fluorescence spectrophotometer was purchased from Shimadzu Corporation (Kyoto, Japan); H1 Multi-functional microplate reader was purchased from Berthon Instrument Co., Ltd. (Newport, RI, USA); EN 3700 Nano Laser Particle Size Analyzer was purchased from Malvern Panalytical Ltd. (Malvern, UK); JEM-2100 transmission electron microscope was purchased from Japan Electron Optics Laboratory, (Akishima, Japan); precision pH meter was purchased from Thermo Orion Instruments Inc. (Waltham, MA, USA); and thermostatic shaking metal bath was purchased from Thermo Fisher Scientific Inc. (Waltham, MA, USA).

### 3.2. Preparation of AuNPs

AuNPs were prepared by the citrate reduction method [31]. The conical bottle was soaked in aqua regia for 24 h. The 100 mL 2.5 × 10^−4^ mol/L HAuCl_4_ was heated to boiling in a magnetic agitator. After boiling for 30 s~1 min, 2.5 mL freshly prepared 10 g/L sodium citrate was quickly injected into the boiling solution under intense agitation. It continuously boiled for approximately 15 min until the color turned orange and no longer changed. Then, the solution was cooled to ambient temperature under gentle speed stirring and was stored at 4 °C.

### 3.3. Preparation and Functionalization of Apt.-SNA

The previous research conducted by our group discussed the cryogenic preparation conditions for DNA-modified AuNPs, and, therefore, this study has adopted the relevant parameters [32]. Firstly, primer DNA was activated in 10 mmol/L TCEP at room temperature for 1 h to expose sulfhydryl groups. After reduction, 0.1 g/mL PEG20000 (final concentration, 0.005 g/mL), 1× PBS solution, and AuNPs solution were added to the primer solution. The final concentrations of AuNPs and DNA were 2 nmol/L and 1 μmol/L [33], respectively, ensuring that each AuNP was fully coated, and resulting in stable DNA–AuNP conjugates. After thoroughly mixing, it was frozen at −20 °C for 2 h. After thawing, the BSA solution with a final concentration of 1% was added to block the remaining active sites on the AuNP surface. After incubation for 1 h, the mixed solution was centrifugally washed 3 times under 15,777× *g* for 15 min.

A total of 20 μL 100 mmol/L ME was added to 20 μL washed SNA solution, and the PBS buffer was supplemented to 100 μL. It was slowly shaken at 37 °C for 18 h. After centrifugation, the supernatant was taken to measure fluorescence at 489 nm/520 nm. Then, the fluorescence standard curves were established under the same conditions, and the DNA load was calculated [34].

### 3.4. Bacterial Culture and Preparation of a Standard Curve for Colony Counts

In the biosafety cabinet, bacteria were inoculated into LB liquid medium and cultured at 37 °C for 24 h (enterica serovar *S*. Typhimurium, *E. coli*, and *S. aureus* were cultured at 37 °C, while *P. fluorescens* was cultured at 28 °C). The overnight-shaken bacterial culture was serially diluted with PBS buffer and subjected to plate counting. PBS solution was used as a blank control to measure the absorbance of the bacterial suspension at 600 nm, constructing an absorbance-colony count standard curve. The bacterial culture was sterilized at 121 °C for 15 min, cooled, and washed three times by centrifugation at 2739× *g* for 5 min using PBS buffer. Finally, the bacterial suspension was stored at 4 °C.

The fluorescence intensity of blank wells (containing only PBS buffer) was measured to account for background fluorescence. The background signal was subtracted from the fluorescence readings of all experimental wells to obtain the net fluorescence intensity (F_net). The net fluorescence intensity was calculated using the following formula:

F_net_ = F_sample_ − F_blank_, (1)

Because the R^2^ of the linear equation obtained in this study is small, the LOD is calculated by substituting the standard deviation of the blank measurement into the linear regression equation

### 3.5. Feasibility Verification

The bacterial solution cultured for over 24 h was aliquoted into two 15 mL centrifuge tubes (10 mL each) and centrifuged at 4 °C and 2739× *g* for 5 min. After washing twice with PBS, the bacterial pellets were resuspended. The bacterial solution was diluted 10-fold to measure the absorbance and calculate the concentration, yielding 20 mL of bacterial solution at 10^9^ CFU/mL. A 10 mL aliquot was sterilized by autoclaving (121 °C, 15 min), while the remaining 10 mL is a solution of live bacteria. Then, 8 mL each of the live and dead bacterial solutions were ultrasonicated at 200 W for 10 min (5 s on, 5 s off). The Apt.-SNA probe was diluted 10-fold (20 μL in 100 μL after PBS washing). A mixture of 20 μL probe, 20 μL bacterial solution (or lysate, blank control, etc.), and 60 μL PBS was incubated at 37 °C for 2 h. DNase I was used to digest the probes, and fluorescence intensity was measured at 489 nm/518 nm.

### 3.6. S. Typhimurium Detection

A total of 100 μL of *S*. Typhimurium antibody solution (prepared in coating buffer) was added to the microplate and incubated with shaking at 37 °C for 2 h. After coating, each well was washed three times with 300 μL of washing buffer. Then, 300 μL of freshly prepared 5% skimmed milk powder solution was added and blocked at 37 °C for 1 h. Following washing, 100 μL of bacterial solution (prepared in dilution buffer) was added to each well and incubated at 37 °C for 1 h to allow binding of the bacteria with the antibodies. It was then rewashed, 100 μL of Apt.-SNA probe was added, and the fluorescence intensity was measured at 489 nm/518 nm after incubating at 37 °C for 2 h (Step 1). The plate was washed, and 100 μL of DNase I solution (prepared in 1× reaction buffer) was added. After incubating at 37 °C for 1 h, the fluorescence intensity was measured at 489 nm/518 nm (Step 2).

### 3.7. Sample Analysis

Canned pickled pork stewed bamboo shoot soup from Zhejiang Shanya Food Co., Ltd. (Wenzhou, China) was purchased as the food sample. The soup was filtered and sterilized. The newly cultured viable *S*. Typhimurium were serially diluted to 10^5^, 10^4^, and 10^3^ CFU/mL with sterilized soup, while the dead bacteria were diluted to 10^5^ CFU/mL. The samples were pre-enriched at 37 °C and 3944× *g* for 8, 16, 24 h. The samples were centrifuged and washed twice with PBS solution. The concentrations of viable and dead bacteria were determined by the plate counting method and the new fluorescence ELISA method.

## 4. Conclusions

In this study, a fluorescent immunoadsorption strategy based on aptamer-modified gold nanoparticles was developed to detect viable and dead *S*. Typhimurium rapidly.

Traditional methods for detecting pathogenic bacteria rely on plate culture techniques, which involve steps such as enrichment culture, biochemical identification, and serotyping. These methods have drawbacks, such as long detection cycles and insufficient specificity. Nucleic acid amplification methods, including polymerase chain reaction (PCR) and loop-mediated isothermal amplification (LAMP), are also commonly used for bacterial detection. These methods offer high sensitivity but are complex to operate, requiring advanced instruments and skilled personnel. Traditional sandwich ELISA, which is based on colorimetric principles, reduces the detection time, but requires the use of dual antibodies, thus resulting in higher detection costs.

Our scheme can eliminate the long and complicated bacterial culture process and high equipment costs. It is simple to operate and can be completed in one day. Moreover, it has good universality, and the detection of different foodborne pathogens can be realized by changing the antibody and aptamer sequences. Although this method requires the target bacteria to produce extracellular DNase, numerous studies have shown that various human pathogens, such as *S. aureus*, *Haemophilus influenzae*, and *Streptococcus agalactiae*, are capable of secreting extracellular DNase [20]. The method is also theoretically suitable for other salmonella-contaminated food substances, such as meat and eggs, as long as the appropriate pre-treatment steps are included.

However, this method still faces challenges in accurately quantifying bacterial concentration, and the relatively high detection limit necessitates the pre-culturing of samples. Future optimization of this protocol could focus on reducing the detection limit through strategies such as modifying the aptamer sequence, incorporating multiple aptamers into the probe, or integrating electrochemical signal detection.

## Figures and Tables

**Table 1:** Detection of *S*. Typhimurium in soup samples.

Enrichment Time	Added Viable Concentration (CFU/mL)	Added Dead Concentration (CFU/mL)	Results of Plate Counting Method (CFU/mL)	Measurement Results of Step 1 (CFU/mL)	Measurement Results of Step 2 (CFU/mL)
8 h	10^4^	10^5^	8.4 × 10^6^	1.02 × 10^6^	3.63 × 10^5^
16 h	10^4^		8.6 × 10^7^	6.84 × 10^7^	2.03 × 10^6^
	10^3^		5 × 10^7^	1.36 × 10^7^	7.1 × 10^5^
24 h	10^4^		3.7 × 10^10^	1.44 × 10^9^	1.29 × 10^6^
	10^3^		9.1 × 10^8^	3.68 × 10^8^	1.57 × 10^6^
	10^2^		1.4 × 10^8^	5.28 × 10^8^	1.35 × 10^6^

**Table 2:** DNA sequences employed in this work.

Name	3′ Modified Group	Sequence (5′ to 3′)	5′ Modified Group
Apt.	HS	TAT GGC GGC GTC ACC CGA CGG GGA CTT GAC ATT ATG ACA G TTTTTTTTTT	FAM

## Data Availability

The data presented in this study are available on request from the corresponding author.
